# Conflicts Between Women's Religiosity and Sense of Free Will in the Context of Elective Abortion: A Qualitative Study in the Worst Period of Italy's COVID-19 Crisis

**DOI:** 10.3389/fpsyt.2021.619684

**Published:** 2021-08-02

**Authors:** Ines Testoni, Nicoletta Finco, Shoshi Keisari, Hod Orkibi, Bracha Azoulay

**Affiliations:** ^1^FISPPA Department, University of Padova, Padova, Italy; ^2^Faculty of Social Welfare and Health Sciences, Emili Sagol Creative Arts Therapies Research Center, University of Haifa, Haifa, Israel

**Keywords:** abortion, grief, religion, COVID-19 pandemic, qualitative research

## Abstract

This qualitative study considers the relationship between abortion, bereavement, and the effects of the COVID-19 lockdown nine women who had undergone an elective abortion, which is voluntarily termination of a pregnancy at the woman's request. These women were interviewed in three time points (1 month, 6 months, and 1 year after the event) to consider the possible evolution of their experience. The third phase was concurrent with the COVID-19 pandemic and particularly with Pope Francis's Easter declaration against abortion. All the interviews were conducted and analysed through qualitative research in psychology. Results showed that the abortion experience led to physical, relational, and psychological suffering, similar to perinatal grief. Participants were non-practising Catholics and religiosity did not help them to overcome their sorrow. Though religiosity is a possible resilience factor in other stressful conditions, in this case it is a factor that aggravated suffering. Finally, we discuss the difficulties experienced by Catholic women who choose to have an abortion and assert the necessity of psychological and spiritual interventions to support these women.

## Introduction

Elective abortion (i.e., the voluntary termination of a pregnancy at the woman's request), is the cause of significant ideological and social controversy and may cause psychological distress for some women (1). This issue involves many countries because of ideological arguments between the pro-life and pro-women's rights perspectives. From the pro-women's rights point of view, the legalisation of abortion is considered to be an important accomplishment by the second-wave feminist movement that introduced the concept of “reproductive freedom” and advocated for abortion access as a civil right (2). In this regard, the World Health Organisation (WHO) (3) assumes the perspective of the International Conference on Population and Development (ICPD) (4), affirming that

every individual has the right to decide freely and responsibly—without discrimination, coercion and violence—the number, spacing, and timing of their children, and to have the information and means to do so, and the right to attain the highest standard of sexual and reproductive health. The access to legal, safe and comprehensive abortion care, including post-abortion care, is essential for the attainment of the highest possible level of sexual and reproductive health.

Although in most Western countries, the legitimacy of abortion is now fairly well-established and the principle of women's self-determination is sufficiently respected, abortion is still stigmatised which may cause significant psychological distress (5–7). Some religiously oriented pro-life attitudes contribute to the spread of social hostility toward women who have elective abortions (8–11).

Scholars have widely shown that religious individuals may manage their grief and mourning more effectively than those who are not religious (12, 13). However, as indicated in the literature (14), religions are often a significant cultural factor in subordination of females. This is an important concern because, in the case of elective abortion, religion may exacerbate psychological difficulties if or when this choice causes grief and bereavement. The present qualitative study examines whether elective abortion causes grief and mourning and how religion may provide support or be detrimental to women who voluntarily have an abortion. In addition, this study discusses some implications inherent to the representation of the foetus and addresses how cultural factors may contribute to an increase in potential mourning.

## The Representation of the Foetus as a Person

One of the ideological positions of the pro-life movement that may cause psychological distress to women who elect to have abortions is the representation of the foetus as a person, from which the discussion of foetal rights derives. “Fetal rights are the moral or legal rights of the human fetus under natural and civil law. This concept includes not only abortion but also any issues of maternal misbehavior that may damage the fetus” (15). Indeed, the American Convention on Human Rights (ACHR) (16) affirms that foetal right to life begins from the moment of conception, whereas the European Court of Human Rights (ECHR) (17) contends that Article 2 of the European Convention on Human Rights does not extend to abortion. However, while European law generally considers the rights of a foetus to be within the mother's rights, ACHR conventions consistently the foetus as a person (16).

Foetal rights viewpoints are influenced by religious perspectives and by the way religions conceptualise “ensoulment” (i.e., the infusion of the soul into the human body) as a sign of becoming a person. This concept is partially shared by Eastern religions and Abrahamic religions (i.e., Judaism, Christianity, and Islam), underlining how a foetus becomes a person only after ensoulment. Members of the Hindu religion, for example, believe that the beginning of personhood coincides with the occurrence of reincarnation at the moment of conception. Thus, even the earliest version of a human foetus deserves protection and respect, but priority is given to the alleviation of suffering of living adult humans (18). The Islamic religion does not provide a clear answer about the time of ensoulment related to conception. Islam respects the foetus because of its potential to grow into a human being, but it does not consider the foetus to be a person, thus distinguishing between actual life and potential life (19). Both Islam and Hinduism emphasise the need to respect and protect the respect and protect thefoetus, but these religions also maintain that abortions can be performed under risky circumstances for the mother. According to the Jewish faith, a foetus does not count as a life and has no legal status until it emerges from the mother's womb and lives for at least 30 days (20). Judaism respects the foetus, as it has the potential to become a human being, but according to this religion, the foetus is not considered a living being, and therefore, the life of the mother takes precedence. In risky situations where the mother faces a clear, life-threatening danger by continuing the pregnancy, Jewish law states that a medical abortion must be performed to save the mother's life because she is considered to be a person while the foetus, until the time of the birth, is not (21).

Regarding Christianity, the current position of the Catholic Church considers human life to begin at the time of conception. Accordingly, the Catholic Church believes that an early foetus is a person and has the same legal rights. However, this belief did not always represent the position of the Catholic Church. This issue continues to be discussed in bioethics, especially concerning artificial insemination, a practise to which the Catholic Church remains opposed (22). In this context, a prominent Catholic theologian, Thomas Aquinas, is considered fundamental. According to Aquinas, one must not confuse the “vegetative soul,” which characterises all forms of living plant and animal matter, with the “rational and volitional soul,” which differentiates humans from animals. As indicated in the first part of Aquinas's *Summa Theologica* (23), the rational soul has supernatural origins and is infused by God into matter, the human body (ensoulment). Accordingly, without the rational soul, a foetus is not considered to be a person and is therefore not equal. However, since the seventeenth century, the belief put forward by Aquinas was not sustained; other attitudes pervaded the Catholic belief, and this novel attitude positions the rights of a foetus equal to those of a person (20).

## Psychological Problems Related to the Representation of the Foetus as a Person

The literature on elective abortion is not as extensive as that on perinatal grief, and the findings are mixed (24, 25). The literature on mental health problems following an elective abortion is often contradictory (26), whereas a report by the American Psychological Association (APA) suggests that “women who are denied an abortion are more likely to initially experience higher levels of anxiety, lower life satisfaction, and lower self-esteem compared with women who received an abortion” (27). Existing literature on potential grief following an elective abortion is scarce, and most studies indicate that the majority of women who have elective abortions do not experience significant emotional or mental health problems (28, 29). However, some scholars have identified a subgroup of women who are negatively affected by this experience (1, 24, 30–32), particularly young women (33). Scholars explored the possibility that an elective abortion has the potential to elicit a short-term grief response (34). However, this area still requires research to identify which phenomenon causes this effect and which women are at greatest risk (25). Furthermore, grief response should be acknowledged and supported (34).

In the area of research about stillbirth, literature has already discussed this as a form of natural abortion that may cause severe and complicated grief because the loss is involuntarily, and the pregnant women who want to have a child already represents the foetus as a baby (12, 13, 35, 36). The representation of the foetus as a child is at the basis of the idea that a foetus is already a person. When a being who is considered a person dies, this death may cause grief and bereavement, as in the case of stillbirth (37, 38). The representation of the embryo/foetus as a baby who is independent of the pregnant woman has been developing since the twentieth century (37, 39). As shown in existing studies on stillbirth, from the perspectives that aim to define and consider the foetus as a child, a particular sense of suffering derives.

Reproductive choice line studies have emphasised the personification of the foetus and specifically blame the social communication that assumes a *foetus-centric* position. In particular, these studies condemn the representation of women's perpetual mourning caused by the loss of their unborn children (40, 41). In this perspective, the centrality of the foetus passes through the description of grief that dominates accounts of abortion across varied and opposite narrations. As Millar underlines (42), the recurrence of foetus-centric grief throughout the media is distressing to women because the emotions convince pregnant women to be already mothers to the autonomous “children” within their wombs. Furthermore, this form of communication represents the abortion experience to be morally problematic and harmful to women (40, 41). Similarly, for Ursula Barry (43) foetus-centrism is an ideology that became pervasive in Ireland since it was assumed by the Irish Constitution in 1983. Furthermore, the Dublin Declaration on Maternal Healthcare, achieved by pro-life activists in 2012, states that abortion is never medically necessary, even to save the life of a pregnant woman (44). In Ireland, anti-abortion discourses used brutal imagery and violence of language before the legal permission under certain conditions was approved by law in 2018 (43). According to Barry (43), the social condemn causes the recourses of medical tourism (45), and underground abortion (46, 47).

The situation in Italy is somewhat similar because Catholicism maintains that life is an unassailable good and that “deliberate and direct killing, however it is carried out, of a human being in the initial phase of his existence, between conception and birth” (48) is tantamount to murder. In fact, this action is considered to be a particularly serious mortal sin (49). The effect of the Catholic Church's influence on the management of the Italian health system is apparent because, despite Law 194/1978 that officially gave Italian women the right to obtain a legal abortion in the first 3 months of pregnancy, abortion is almost impossible to obtain in public hospitals. This is because Article 9 of that same law permits the conscientious objection of medical personnel. This professional freedom has created serious obstacles to the effectiveness of how Italian women can exercise their right to abortion. In 2018, conscientious objection by gynaecologists was 69%, compared to 68.4% in 2017 (50), and Elena Caruso (51) reported several aberrant cases of medical conduct that resulted in the death of the pregnant woman. Literature (52) has also widely underlined how, as suggested by the European Committee of Social Rights (ECSR) (53), balancing women's right to health and medical personnel's right to conscientious objection results in the reduction of legal surgeries/interventions and more women finding recourse in clandestine abortions. As the United Nations (UN) Human Rights Council working group on the issue of discrimination against women in law and practise (54) emphasises, the problem of clandestine abortions is quite significant because “unsafe abortions cause the deaths of some 47,000 women each year and a further five million suffer some form of temporary or permanent disability.”

From a psychological point of view, because the nature of unwanted pregnancy and subsequent abortion causes women to perceive a lack of freedom regarding the control of their lives or bodies, women often feel compelled to keep their choice a secret, or they feel delegitimized in their suffering (55). Since the right to access safe abortions is influenced by ideological arguments, the possibility of grief—even in a subgroup of women who had an elective abortion and for some reason remained affected by the social representation of the foetus as a child—is not recognised. The disenfranchisement of this possible grief is due to the fact that it is considered negative both by pro-life movements and by pro-woman line movements (56–58). The most significant problem, from a psychological point of view, is that when grief is delegitimized, it can degenerate into prolonged grief (58).

Indeed, the problem of potential grief following an elective abortion is trapped in ideological and cultural arguments between foetus rights vs. women's rights (59, 60): from a pro-life perspective, a woman who has an abortion is considered a murderer; from a pro-woman line perspective, she should not suffer because the foetus is not a person and because any potential grief is the result of the media's influence to construct the personification of the foetus.

## Materials and Methods

### Context and Aims

The present study has adopted the socio-cultural perspective, starting from the report of the APA Task Force on Abortion and Mental Health (61). From this point of view, women's experiences with abortion are shaped by negative social and cultural messages; however, it would be possible to promote women's emotional well-being (1, 61). This study was born from the idea that adequate psychological support can help those who elect to have an abortion so that women can resolve their conflicts or possible grief, becoming aware of the cultural complexity that characterises the practise of abortion (62).

This study was specifically inspired by the clinical experience of researchers who had several cases of women, especially one, who suffered from complicated grief due to an elective abortion (63, 64). Subsequently, the study analysed the experiences of women who elected to have abortions and thereby understand the psychological, emotional, and relational aspects involved in their possible distress or grief. In the further conviction that it is important to consider the elaboration of grief and its complexity in elective abortion, which may be similar to the suffering caused by stillbirth, the present research paid particular attention to the nature of this possible conflict.

This research assessed participants' abortion experiences at three distinct points in time—1 month, 6 months, and 1 year following each participant's abortion—in order to examine the persistence of their potential mourning. Coincidentally, the final phase of this research occurred during the most severe period of Italy's COVID-19 lockdown. Furthermore, around that time, Pope Francis publicly condemned abortion, listing it among the worst sins in the world, along with war and arms trafficking (65). This condemnation by the Pope Francis resulted in surprise because it was a particularly difficult period, especially for women. Indeed, the lockdown conditions were exacerbating the conditions of most women, especially those who were victims of domestic violence (66–68), those who lost employment, and those who had to enlarge their commitment to their children at home. His statement seemed to contradict the attitude of his predecessor, Pope Francis, who had previously seemed more tolerant. For example, during the Jubilee of 2016, he granted all Catholic priests the authority to absolve women who had had abortions of their sins (69). The pope's declaration could have negatively affected women who were also stressed because of the lockdown.

### Participants and Procedure

In line with the thematic analysis (TA) procedure (70), the study followed a qualitative research-in-psychology design that utilised in-depth interviews (71) concerning the existential and personal dimensions related to abortion. The invitation to participate in an interview was presented by the medical staff before the intervention. In 2019, roughly 600 women who received an abortion at one of three hospitals in northern and central Italy were invited to participate in the study; 21 initially agreed to participate, but only nine took part in the telephone interview. The average age of participants was 29 years (*SD* = 5.40). Six participants had no children, one had two children, and two had one child. Five participants were living in stable relationships, one was single, and three reported being in unstable relationships. Seven participants selected a pharmacological abortion (RU-486 pill) and two chose surgery. The average gestational age at the time of abortion was 5.8 weeks. All participants reported that they had received a Catholic education and that they identified as Catholic, despite not actively practising the religion.

The number of participants was small, due to the sensitivity of the issue, although it was possible to proceed because qualitative research makes no inferential or generalisation claims, so even small groups can be analysed (72–74). Because the study attempted to make sense of how women think through their lived experiences during and after their abortion and during the COVID-19 pandemic, the semi-structured interviews were conducted by Interpretative Phenomenological Analysis (IPA) (75, 76). The participants were then asked whether they felt comfortable enough to support the research and were required to sign an informed consent form before proceeding. They were also asked to confirm their consent after all the interviews. We preferred to use a qualitative methodology to track women's individual perspectives and narratives. Furthermore, intending to carry out longitudinal research, we did not want to utilise standardised tools to avoid the risk of any learning related to the questionnaires used. This may have been the reason why such a significant number of women preferred not to participate. It is unclear if we could have achieved a greater level of participation if a simple questionnaire had been presented.

The interviews were conducted in three timepoints over the course of a year: first-phase interviews occurred in April–May 2019; second-phase interviews occurred in September–October 2019; and third-phase interviews occurred in April–May 2020, when the COVID-19 outbreak was most severe in Italy. Interviews lasted about 1 h each. Interviews were audio recorded and then transcribed verbatim in Italian (75, 77).

The interviews elicited respondents' horizons of meaning, as is characteristic of IPA, and the analysis attempted to recognise the main themes that were common among interviewees rather than within each of them (78). Similarly, as in other studies that integrate two methodologies (in this case, TA and IPA) (79–81), the texts underwent an analysis to identify similarities and specificities across all the narratives (82). In this process, thematic patterns were identified using Atlas.ti software (83). The analysis performed by the interviewer and supervisor followed six fundamental phases: conducting preparatory organisation; generating categories or themes; coding data; testing emerging understanding; searching for alternative explanations; and writing up the report (70, 84). To verify the correctness of the analysis and interpretive procedures adopted by the interviewer and the supervisor, two other members of the research team worked on the texts until agreement was reached among all the researchers. Common patterns and emergent themes were identified to illustrate convergences and specificities among all participants' answers through a systematic comparison across the texts. The connexions were identified and interpreted through abstraction, which allowed the researchers to recognise the main emergent themes (85). Two of the authors jointly developed a temporary codebook using the transcripts and attempted to ground each code in the participants' narrated experiences. Together, in an iterative process, they extracted codes and identified sentences that contained a single theme. After this, the coding was organised to refine and reduce the various themes to produce inclusive main themes. All differences of opinion were resolved through discussion until the codes were agreed upon unanimously. The codes were assigned descriptive labels that were consolidated into themes, then reviewed and revised several times through discussion. Finally, a consensus was reached with additional, supervising authors. The flexibility of this approach allowed for unexpected issues to emerge from the narratives without the use of a structured hypothesis guided by the literature (86, 87).

The research followed APA Ethical Principles of Psychologists and Code of Conduct and the principles of the Declaration of Helsinki, so participants were told in detail about all the objectives of the research and the methodology of analysis used. They were asked permission to record the conversations, to transcribe their answers, and to analyze their contents to study the phenomenon. We guaranteed to anonymize the contents of the obtained texts and only those who gave written and signed consent participated in the research. All the names cited below are fictitious, and the quotations have been slightly changed to prevent any possibility of identification of the participants. The study was approved by the Ethics Committee for Experimentation, University of Padua (n. 8F57FABE51217661765620C2987CE97C).

## Results

The analysis of all the texts revealed three prevalent themes. The first was “pregnancy and abortion,” which distinguished the first phase and involved the following main issues: abortion as a traumatic event; abortion as liberation; guilt; forgiveness; various reasons for pharmacological abortion (maintain consciousness, less invasive, respect the embryo); various reasons for abortion in general (socioeconomic or relational reasons, or the view that motherhood is a choice); and awareness of having killed. The second prevalent theme was “the relationship with the partner in the context of loss and mourning,” which characterised the second phase and involved the following main issues: conclusion of the relationship with the partner; blaming the partner; strengthening of the relationship with the partner; loss of maternal desire; and trauma when seeing the embryo. The third prevalent theme was “the COVID-19 experience in the context of the pope's declaration, fault, and avoidance,” which typified the third phase and involved the following issues: COVID-19 experience and anniversary; religious crisis and failure of the pope's intervention.

All the names cited below are fictitious and the quotations have been slightly changed to prevent any possibility of identification of the participants.

### First Theme: Pregnancy and Abortion

The first theme corresponded to the first phase of the research. Most of the participants described the discovery of their pregnancy as traumatic, as demonstrated by Arianna:

It was very traumatic for me to find out that I was pregnant. Indeed, I used the morning-after pill and was convinced that I had solved the problem, and I didn't know that this kind of intervention might not work.

This was also the case for Eleonora, who used the morning-after pill, but also found that it did not work. Because she could no longer trust chemical remedies, she opted for a surgical abortion:

Yes, it was traumatic to discover that this medical device may not work. In addition, we always used a condom and never had any problems. I don't want to have a child before marriage or before I am ready for this step.

For almost all participants, the shock was accompanied by anguish and guilt, as Franca clearly expressed:

I experienced a period of anguish. I felt guilty from the very first moment. I was moving house and I also had to change my job. Nothing was stable in my life, and I had economic problems. Finally, I was afraid of losing my partner because I had an abortion.

Michela chose to have an abortion based on the will of her partner:

Everything was already very difficult with my partner. When I found out that I was pregnant, I discovered that he didn't want to be a father. He doesn't even care about the child I had from a previous relationship. I didn't want to tell my future son that he has no father because his father didn't want him.

The use of the RU-486 pill helped some of the participants to manage the initial trauma of their pregnancy, as in the case of Rebecca:

Pregnancy was traumatic because I was sterile. I perceived that I was supposed to use RU-486 because abortion was my responsibility and I had to be aware of it. I had the impression that, if I had done the surgery under anaesthesia, I would not have been conscious. I preferred to use the pill because it seemed more natural. I wanted to respect the embryo and act as if it were a natural miscarriage.

This narrative was similar to that of Sonia:

As traumatic as it was, I always thought that the first person to decide is me, regardless of my partner. I wanted to fully undergo this experience and become aware of what it means. That's why I chose the pill instead of surgery.

Two Catholic physicians chose the same method for different reasons. Caterina chose to use the RU-486 pill because “it is a less invasive method. It creates fewer problems because it avoids anaesthesia, and you don't have to suffer all the consequences of scraping.” Cristina did not experience the discovery of her pregnancy or the use of the RU-486 pill as particularly traumatic. She explained, “This experience was not particularly traumatic; the use of the pill caused a stronger, more liberating menstruation.”

Contrary to Cristina, almost all the other participants found the experience of abortion to be difficult. Federica testified,

It was all very traumatic, and I reacted very badly. I perceived every moment, from the discovery of the pregnancy to the failure of the morning-after pill to the hospital, as a terrible experience. I chose surgery because I didn't trust the pills anymore. I didn't want to have a child and that's why I had an abortion, but I also didn't want to have that experience.

Michela said:

After the pill, my hands were shaking—I couldn't hold a glass of water. I spent half an hour in an empty waiting room. They left me completely alone. I was suffocating with anxiety, but also with rage. (…) I wished my partner were dead, not my son. That's the only truth.

Similarly, Rebecca said:

When I began to feel the detachment and the first bleeding, I felt myself sinking. I realised that the pill I had taken was doing its job, and I felt that I was in an abyss. The most terrible thing was that I felt like a mother that couldn't protect the creature I was carrying due to the pill I had taken. (…) After this moment, I no longer wanted to be supported, and I wanted to continue to face the situation alone.

Arianna shared:

The closer the day of surgery got, the more I was aware of what I was going to do because my body had changed. I felt guilty, as if I were going to kill a human being. But if I thought about it, I knew that I wasn't killing a person but an embryo. But the last few days have been very, very difficult.

Two participants saw the embryo as it was expelled. Michela reported:

I really saw the placenta. I think it was the placenta. I saw a smooth ball altogether, and I had no more losses—apart from a few small drops. I started crying inside the bathroom. I felt terribly lonely. God might have been there, but it wouldn't have changed anything because I would have been lonely anyway.

Similarly, Cristina said, “When I expelled the embryo, a small embryo, I realised I had expelled my child. The others did not take it away from me, and this made me feel alone in front of this fact.” For Caterina, the representation of “killing” was an important element of this experience:

Unfortunately, I am in too unstable a relationship with my husband, and I didn't feel like having a third child. Too much uncertainty and too much responsibility. I am a believer, and therefore I underwent this experience knowing that I was killing my child. And now I keep thinking that I don't have my baby anymore. It would have been better to lose my husband than to lose my son. Instead, I killed my baby, and that is why he will not be with me as well. Now I often think that it's my fault; I didn't save him.

### Second Theme: The Relationship With the Partner in the Context of Loss and Mourning

The experience of loss and mourning emerged in the second phase of the study. Arianna experienced an episode in which she wanted to leave her partner:

It was his fault that I got pregnant. He could have been more careful. So, I really tried to get as far away from him as possible, and to not have sex with him anymore. After this, he helped me to overcome this feeling, and we found another way to stay together and to deal with this sorrow.

Franca's experience produced the opposite result: “There was a period before and after the operation when I didn't want to see him. I was so sorrowful that, for a while, I thought it was all over for me. Finally, I left him.” For Caterina, the abortion triggered a deep mourning period because she became aware of her fatigue in managing an unstable marital life and a parallel relationship:

I had to face the painful problem of my really unstable marital relationship, and I had to make sense of the parallel relationship with another person. Now, this storey is closed. I'm sorry for both: the abortion and the end of this storey.

Sonia also fell into a deep mourning period, which was characterised by resentment over the past:

I began to ask myself if he could be a life partner or if it was a superficial relationship. I was dealing with an immature man, so much so that, when I told him it was over, he got angry and started screaming that he would have wanted the child. But I don't think he even thought that because he never showed any interest in my pregnancy. After I left him, I started another love storey, and my new partner and I are thinking about having a baby.

Rebecca also fell into a crisis with her partner:

I'm very sorry for the loss of the child. I couldn't forgive my partner for what happened. I kept telling myself that, if I had had a man around me who could take responsibility, I wouldn't have felt compelled to do what I did. Now, this storey is over and I'm just alone. I don't believe in stable relationships, so I just stay that way, with my son.

For Alice, the biggest cause of mourning was the abortion's potential consequences for her future prospects of having a child:

I didn't want to have a child at that time, but I want to have children in the future. My worry is that, since I didn't want the child when it came, he or she won't come in the future when I want to be a mother. It's a thought that has been distressing me constantly.

After the experience of seeing the foetus, Caterina and Michela felt an intense sense of guilt. Perla tried to solve it by asking God for forgiveness: “I was suffering a lot for what I had done. Finally, I went to speak to a priest who understood my problems. In confession, I explained the whole situation to him and obtained absolution.” Similarly, Michela explained:

I also sought comfort in religion. After years of not reciting the Lord's Prayer, I began to pray during mass at a neighbourhood celebration. Then I spoke with a nun. Unfortunately, it did not help me, and my sense of guilt remained the same. I also thought about going to a psychologist. Maybe I should have done it earlier, as I can't get past this blockage. Finally, I asked the son I had aborted for forgiveness, and I hope he has forgiven me. I would like him to be well, wherever he is.

### Third Theme: The COVID-19 Experience in the Context of the Pope's Declaration, Fault and Avoidance

On the contrary, the third phase of the research occurred exactly 1 year after the abortion experience, during the COVID-19 lockdown and Easter time, and straddling Pope Francis I's declaration against abortion. The experiences described are diverse in nature. Caterina suffered badly during this period because she is a doctor and had to separate herself from her two children. She often thinks back to the experience of abortion, realising how difficult it was and how difficult it is now to manage this thought together with COVID-19:

Abortion took me away from others. Now, COVID-19 accentuates this difficult experience. It is as if I can no longer return to normal. I have all this experience of death, remembering every day what happened to me the year before. I keep thinking back to the moment I found out I was pregnant, to the decision to have an abortion, where I made the call to make the appointment. I went back voluntarily to the place where I had the abortion. I'd been there a million other times before for work, but it never really hit me. This time, I looked at it another way. I gave myself permission to remember. Obviously, all this hurts. My psychologist says I think I've committed murder. Maybe or maybe not, because I'm Catholic, but I know I did something very serious. Even if I had the right, I can't pretend it didn't happen.

Although Cristina is a non-observant Catholic, she judges the pope's intervention as unsuccessful:

It is not fair to absolutize this. I really disagree. Women must be left free to choose. It is not fair to compare abortion to murder, to the sale of weapons, to the violence of war. It is universally right to urge men not to kill, but abortion cannot be compared to it. Indeed, I agree that one should not abort lightly, but I did not kill a man with a gun. An agglomeration of cells cannot be compared to a sentient, conscious, living human being. For me, it was a matter of choosing life—my life—and in similar situations we must leave the woman free to choose.

Rebecca also underwent the COVID period remembering each day what had happened the year before:

COVID's nightmare came exactly 1 year after my abortion. Everything became more difficult because of the pandemic, and I inevitably thought that, if I had carried on with my pregnancy, I would have found myself in unmanageable conditions. I have had to face serious practical and economic problems and also loneliness. If I had not made that choice, I would have found myself even more lonely, facing enormous responsibilities without any help. (…) I'm Catholic, and the fact that the pope compares me to warmongers, to murderers and to those who sell weapons baffles me. Although I'm pained by the choice I made, and although I consider it to not be a good thing, I certainly don't think I can be associated with an assassin, someone who kills people for money. The pope's way of thinking is not Christian, it is Catholic. It is a way of thinking that does not enter into the lives of women and their worries. I agree that abortion is horrible, that it is an act against nature, but the women who chose abortion must be supported and understood, not condemned as assassins.

Sonia handled the issue of abortion more thoroughly with her new partner:

During pandemic, the time spent living with my new partner has increased. We've had more time to be together and even to sit at the table, facing each other, in order to talk about important personal things, and this is important. It occurred to me (that) it was the anniversary. During the lockdown, I had time to rethink that whole experience. It's not right to inflame the matter like the pope did.

Arianna dreamt of the child she could have had if she hadn't had an abortion:

Once I dreamt that I was in a situation where there was an already-big baby that I was raising as my own, but he wasn't mine. I thought that the child was growing up in another dimension that we don't know, where we go after death. I don't think it's “heaven,” but I hope it's a place where we're going to be good anyway.

Similarly, Cristina said, “He still exists somewhere and is preparing to be born again in the future. I think it is a person who will come here at another time.” Alice, however, is uncertain: “I am a Christian, and I believe that my religion says that children go to heaven, but this was nothing more than an embryo. I don't know what an embryo is and what existence it can have in heaven.”

In Arianna's opinion, the best way to overcome the trauma is to eradicate it from her mind:

My mind wanted to eliminate this experience (as this is), the best way to do it and prevent me from thinking about it, because it is painful. I didn't want to talk about it with anyone. I asked something to a woman who had had the same experience to understand if my body's reactions were normal. She was the only person I talked to.

Eleonora shared a similar perspective:

Even though I needed to talk about it, I didn't, and I faced this experience alone, without talking about it with anyone. I tried to close up, to not follow the saddest thoughts, and now I no longer feel the need to confide in someone.

The avoidance of abortion-related thoughts and memories was even more explicit for Michela: “I forced myself to not think about it anymore, to erase all this pain. I have rejected everything that has happened to me. I don't even remember what happened or why I did that.” Rebecca said:

I don't tell anyone about it. I try to get through it all by myself, and I realise that I'm getting loaded with so many things to avoid thinking about it because this thought still makes me suffer a lot.

Similarly, Sonia said, “I avoid talking about it now, even with my closest friends. I'd like to erase everything from my brain, remove all this experience.”

## Discussion

Our results are in line with the existing literature on women's reasons for having an abortion. For example, similar to what by Littman, Zarcadoolas, and Jacobs (62) described, “women make hard choices about pregnancy and abortion because they care about raising children well,” assumed that “women have abortions because they need to take care of the children they already have. …Some women feel that by having an abortion now, they will be in a better place to raise children in the future …Sometimes, abortion is the best option we have to protect the long-term health and well-being of ourselves, our families, and our future families” (62). These results are also in line with those of a study conducted by Aléx and Hammarström (88), involving five women, and using a similar research design, in which the experiences of abortion related to financial problems, being too young, and an insecure partnership.

However, unlike all literature that describes abortion as an unproblematic experience, the narrative paths of our participants took three different trajectories: a positive path for one participant, a permanently negative trajectory for five participants, and one worsening path for three participants. Arianna was the only participant who demonstrated a progressive path of improvement and an exit from the initial negative experience. The crisis of her first interview evolved positively in the second, and her third interview indicated that the pope's announcement caused no negative effects. Unlike Arianna, however, Michela, Caterina, and Rebecca maintained a substantial negativity throughout the meetings. Furthermore, for Caterina and Rebecca, the pope's speech worsened their discomfort. Indeed, the reactions to the pope's condemnation were different. In fact, Sonia and Cristina took a strongly critical stance that distanced them from the Church's position. If religiosity is a protective factor for the management of suffering, especially concerning death and mourning, the evolution of Sonia and Cristina must also be considered in some way pejorative. During the first two interviews, the topic of religion was managed in a soft way as not to generate discomfort in the participants. Their answers regarding the topic of religiosity were elusive. On these occasions, all participants stated that they had received a Catholic education and considered themselves generally Catholic but were not practising or actively involved in a parish. In the third interview, the religious issue was unavoidable and also negative. Only one participant, Marzia, became a practising Catholic to resolve her sense of fault and loneliness.

The negative aspects of the abortion experience are multiple, oscillating between trauma, an ambivalent sense of liberation, and a sense of fault, loneliness, and failure.

In the first interview, it was clear that, as with other instances described in the literature (89), our participants found the discovery of their pregnancy to be traumatic, and they portrayed it as an event that made everything uncertain. All reasons for electing to have an abortion involved the restoration of equilibrium, which, in nearly all cases, had been compromised before their respective pregnancies on both the socioeconomic and emotional/family levels. Similar to the results described by Kero and Lalos (90) and Aléx and Hammarström (88), the women's experiences were ambivalent: distressing on one side and liberating on the other. The concept of ambivalence implies a “both-and” rather than an “either-or” process and content (91). In social psychology, ambivalence is described as an attitude that can contain separate positive and negative components (92, 93). Indeed, our participants were ambivalent because they expressed relief while simultaneously experiencing the termination of the pregnancy as a loss that elicited negative feelings of grief. However, despite their suffering, the participants' respective abortions led to increased self-awareness. Thus, this ambivalence can be regarded as not only problematic but also indictive of an openness to the complexity of the abortion issue from a moral point of view. Indeed, participants invoked the principle of self-determination and recognised that it was linked to their freedom (55).

It is important to not underestimate the distress related to this profound ambivalence. Multiple factors caused the negativity of the experience. Among them was the indifference of health-care personnel during the abortion experience, which plunged the participants into strong estrangement experiences, exacerbating the stress accumulated in the pre-abortion phase. Also, for participants who used the abortion pill, the sight of the embryo was highly traumatic. This is perhaps inevitable considering the social representation of the foetus as a child that characterises the Italian culture. The participants reported three primary reasons for choosing a pharmacological abortion: to maintain consciousness, respect the embryo, and select a less invasive method. For some participants, this experience was particularly painful, so much so that Arianna concluded that surgery would have been preferable. There are discrepancies in the literature on this subject. Some researchers argue that pharmacological abortion is not related to psychological distress (94), while other authors claim that trauma can be sustained when women see the aborted embryo (95), as demonstrated in Caterina and Michela's experiences.

Nonetheless, the participants in this study ultimately and consistently characterised their outcomes as liberating. Still, this did not prevent the experience of grief and mourning, especially because of a lack of social support. Previous literature has highlighted how important it is for women in this situation to receive positive support (96). However, this often does not happen because women feel too guilty and ashamed of their conditions to seek support (97). This kind of grief may lead to perinatal suffering, as it fails to neatly adhere to cultural grieving expectations that determine whether and how the suffering of individuals is acknowledged. As illustrated in both literature and the results of our study, abortion precipitates the deterioration of both the source relationship with the partner and, by extension, one's relationship with oneself. Thus, women require psychological support to process the experience as a whole and to manage the resulting suffering (28).

The coinciding religious condemnation of abortion by Pope Francis, which was associated with feelings of guilt, shame, and a lack of social support, likely contributed to the women's avoidance of painful thoughts related to the traumatic experience. All of these feelings certainly contributed to worsening relationships between participants and their partners, which in some cases resulted in the termination of the relationship. Furthermore, the Church's intransigent position on abortion caused participants to experience additional trauma based on religious stigma and the pope's explicit rejection.

From these narratives, we learned that the experience of loss was traumatic for our participants and that the mourning process stimulated a desire to quell related thoughts and feelings of pain, shame, and guilt, given the impossibility of finding social support. This strategy essentially entails a suspension of suffering through processes of removal and negation. Neimeyer points out that overcoming trauma requires a deep awareness of what has been suffered (98), which is achieved by thoroughly reconstructing the existential meanings of the experience of loss (99, 100). Because this awareness was inaccessible to the participants during this study, they seemed more vulnerable to the effects of COVID-19 and the discourse caused by the pope's announcement. The pope's denunciation may have further distanced the participants from any form of support and from a resolution to their mourning. Participants blamed the pope for his unwillingness to understand their situation while also asserting that abortion is a sin. Based on the pope's statement, the Catholic Church does not allow any mediation between these two elements, resulting in a cognitive dissonance from which Catholic women cannot free themselves except through denial and removal. These particular narratives help to understand how previous clinical cases that authors encountered could have developed prolonged and unresolved grief. Catholic women who choose abortion may be particularly susceptible to self-blame, and this suffering may persist in the future, even after many years (16, 101). Though religiosity can greatly assist those who endure the pain of loss and trauma (63, 102), in this case, the pain embodies an additional trauma—that is, feeling rejected by the religion to which one is devoted. As evidenced in the literature, this experience is similar to that of divorced Catholic individuals (103) and of Jehovah's Witnesses who broke away from their original group (104). All of these religious people suffer from cumulative ostracism which generates great psychological distress because of a series of rapid losses. For example, within the couple, there can be the loss of individual identity and the loss of partner support; however, within the religious group, there can be the loss of social identity derived from the religious affiliation, the loss of group support, and for some, even the loss of faith. Thus, the women in this study required support to manage both the trauma of abortion and the trauma of religious rejection. The study of Kero and Lalos (90) confirms that social perspectives legitimise the decision to have an abortion, while ethical perspectives complicate that decision. In such a milieu, feelings of guilt and psychological distress inevitably derive.

The existing psychological literature, in line with the APA indications (61, 105), shows that it is possible to intervene, to offer a positive process of such an experience, giving it meaning. As argued extensively in the National Academy of Medicine report (106), it is possible to ensure that women receive adequate support before and after an abortion and, if engagement is positive, the supportive relationship has a greater chance of effectiveness because women trust the health service. In particular, the intervention of Littman et al. (62) demonstrated that it is possible to introduce women to a “culture of support” by providing them with a positive framing of their experiences. The authors provided validation and information about available support services to sustain women in the reproductive choices they had made and informed women about the services and sources that provided misinformation.

As the UN (54) asserted, it is not by criminalising abortion that its incidence is reduced.

There are no sufficient studies regarding support for the spiritual elaboration of the abortion when it might be experienced. The unique research on this issue by Layer and collaborators (32) showed that in the subgroup of women who had post-abortion grief, characterised by shame and post-traumatic stress disorder (PTSD), a spiritually based group intervention could help to mitigate the negative effects. Indeed, post-intervention measures indicated decreases in shame and PTSD symptoms, and religious motivation was the basis of this activity's participation. An intervention such as this should elaborate upon the difficulties that emerge from the representation of the embryo as a person that perpetuates the perception of having killed a child.

The study of Inglehart (107) shows that from 2007 to 2020, an overwhelming majority of countries became less religious. This decline in belief was the strongest in high-income countries. Inglehart outlines the reasons why this religious decline has been so sudden and systematic throughout most of the world. The most powerful factor that causes this decline in religiousness is closely related to the imperative of maintaining high birth rates. In the past, most societies assigned women the role of producing as many children as possible. While this societal role primarily discouraged abortion, it was also intended to discourage divorce, homosexuality, contraception, or any other sexual behaviour not intended for purposes of reproduction. In the opinion of Inglehart (107), all major religions, including Catholicism, encourage high fertility in periods of high infant mortality and low life expectancy, presenting pro-fertility norms as an absolute and attributing them to the will of God. Throughout history, religions that did not promote the high birth rate as a value gradually disappeared because of the extinction of their followers due to infant mortality and low life expectancy. At present, a higher quality of life and the discoveries of medicine have drastically modified the situation, making these traditional cultural norms no longer necessary. In societies that reach a sufficiently high level of economic and physical security, younger generations have grown up abandoning the norms regarding fertility and the ideologies that absolutize them. The seventh wave of the World Values Survey (WVS) (108), which took place in 2017–2021, indicated that in those countries, secular-rational values are contrary to traditional values and that these societies place less emphasis on religion, traditional family values, and authority. Divorce, abortion, euthanasia, and suicide are seen as relatively acceptable. In this way, cultural representations and attitudes toward gender equality, divorce, abortion, and homosexuality are now rapidly changing.

The situation is the same in Italy (109). Regarding abortion, only 23% of the sample interviewed in WVS-7 considered this choice “never justifiable,” while in WVS-5/2005–2009 (110), the figure was 40%. Similarly, religion resulted in responses of “very important” in WVS-7 (109) from 23% of the sample, while that figure was 34% in WVS-5 (110).

What we want to emphasise here is that religions risk losing credibility because of this issue of not allowing people access to the substantive idea, which is now much more important than reproductive instances; that is, the mourning implied regarding death and the suffering. On the other hand, it is undeniable that literature on abortion may be biassed to some degree by the different ideologies that drive research on the subject. Given the ongoing conflict surrounding abortion, continuing and future research must reflect more widely on the multiple perspectives that inform this issue (111). Moreover, this field still requires more specific research to guarantee better support for women who suffer from this potential and specific form of grief.

## Conclusion

This study confirms that elective abortion may be a traumatic experience that has very negative repercussions for affected women's psychological, physical, and relational well-being. All areas of psychological life are affected, and this suffering may become in some cases very similar to perinatal grief. However, abortion-related grief differs from perinatal mourning with respect to the strong moral-religious connotations attached to this event, which make affected women feel guilty and socially isolated. The participants in this study perceived the pope's public condemnation of abortion during the Easter Vigil—in the worst period of Italy's COVID crisis—as disproportionate to the reality of abortion, and they interpreted it as a testimony of the Church's non-acceptance of their needs and difficulties. Clearly, it is necessary to improve appropriate counselling resources for women who undergo a similar experience, as religiously derived moral guilt and social disenfranchisement may facilitate the future onset of prolonged grief disorder, especially in Italy, where religious attitudes are especially pronounced.

## Study Limitations and Future Directions

Although the study demonstrates the need to make sense of the abortion experience and addresses a serious lack of research and literature on the associated form of grief, the limitations of this research are numerous.

One limitation is the small number of participants. Given the strong condemnation of abortion that persists in Italy, many women decline the opportunity to discuss their experiences. In this article, it may seem that the condemnation of abortion by the Catholic Church is the main reason why women who make this choice are stigmatised. Obviously, this is not always the case, as literature has shown (11, 112), even if in Italy, Catholic culture has a strong influence on both political and cultural life. Although many women were contacted, only nine were interviewed. Therefore, it is likely that a complete account of the phenomenon investigated here cannot be achieved through this small number of participants. What characterised the group may have been the need to give meaning to the abortion experience, and the women who did not respond to the invitation may not have had this need. Thus, it is conceivable that the group of participants was not representative not only because of the small sample size but also because of the specific motivation to participate in this research. Indeed, it was not possible to control the entire process of participant selection and to start with engagement that accounted for reasons why women participated. Future research could begin this process through an invitation during a counselling session in the pre-abortion phase. In our case, only the participants showed that they could or would talk about their abortion experience, and the interview was probably a surrogate for another type of need that could have been handled with a counselling intervention.

Moreover, to avoid making the participants suffer too much, the possible relationship between guilt and religious belief has not been sufficiently investigated. However, future research could focus on this issue and then find suitable strategies to reduce any risk of stress for the participants. A more specific analysis could address the experiences of shame and insecurity related to social desirability. This type of future investigation, which was not carried out in this study, should therefore consider participants' cultural context. The researcher should determine the extent to which their contexts influence the social representations of the role of women and if they are rooted in a religious sense and not only with respect to Christian religions. In fact, the limitations of this study are also related to the fact that women's histories change, and the way their roles are represented also changes according to different contexts. Future research, therefore, will need to focus in greater detail on the analysis of the relationship between the individual, the social context, and the religious culture of reference.

A quantitative multicenter study could also be carried out with measurement scales focused on these issues to be administered to randomised groups throughout the country to verify how some variables such as guilt and stress can change depending on the context of reference. Further future studies could include quantitative and longitudinal surveys to cheque any possible changes in the relationships between religiosity, social desirability, PTSD, and grief in a randomised control trial. This requires employing a more participatory design, thus increasing women's motivation to participate by allowing them to better understand the study's purpose and the usefulness of elaborating abortion-related feelings. The findings highlight the need for future intervention studies on this topic, which could employ experiential approaches such as psychodrama and arts therapies (113–116) to help participants further process their loss and foster their empowerment.

From the point of view of future perspectives of clinical and psychosocial intervention, the present study suggests that counselling and support services should be provided and activated, capable of not imposing a specific ideology in order to free women from guilt, even when they are Catholic. This type of counselling serves to protect the quality of their lives and to allow them to maintain a deep and positive relationship with their spirituality, since this is a very important aspect for all people, especially when they must face difficulties, illness, and death.

## Data Availability Statement

The raw data supporting the conclusions of this article will be made available by the authors, without undue reservation.

## Ethics Statement

The studies involving human participants were reviewed and approved by Ethical Committee for the Psychological Research of the University of Padova. The patients/participants provided their written informed consent to participate in this study.

## Author Contributions

IT: research design and project planning, supervision of the research, analysis of the texts, methodology, and article writing. NF: project planning, interviews, analysis of the texts, and article writing. SK, HO, and BA: article writing. All authors contributed to the article and approved the submitted version.

## Conflict of Interest

The authors declare that the research was conducted in the absence of any commercial or financial relationships that could be construed as a potential conflict of interest.

## Publisher's Note

All claims expressed in this article are solely those of the authors and do not necessarily represent those of their affiliated organizations, or those of the publisher, the editors and the reviewers. Any product that may be evaluated in this article, or claim that may be made by its manufacturer, is not guaranteed or endorsed by the publisher.
